# Supplementary material for: PD-L1 Is Involved in the Development of Non-Hodgkin’s Lymphoma by Mediating Circulating Lymphocyte Apoptosis
Source: Vaccines (Basel). 2023 Sep 11;11(9):1474. doi: 10.3390/vaccines11091474 (PMC10538143; doi:10.3390/vaccines11091474)
Supplement: Supplementary file 1 [file vaccines-11-01474-s001.zip › vaccines-2226306-supplementary.pdf]

Table S1. Correlation between percentages of annexin-V$^+$ cells and the significant variables in NHL patients. *$p < 0.05$; **$p < 0.001$.

	**Annexin-V$^+$, %**	
	**r**	***p***
Annexin-V$^+$, %		
Annexin-V$^+$PDL-1$^+$, %	0.791	<0.001**
Annexin-V$^+$PD-1$^+$, %	0.835	<0.001**
CD3$^+$, %	0.853	<0.001**
Annexin-V$^+$CD3$^+$, %	0.890	<0.001**
PDL-1$^+$CD3$^+$, %	0.593	0.006*
PD-1$^+$CD3$^+$, %	0.805	<0.001**
CD3$^+$CD4$^+$, %	0.594	0.006*
Annexin-V$^+$CD3$^+$CD4$^+$, %	0.489	0.029*
PDL-1$^+$CD3$^+$CD4$^+$, %	-0.11	0.645
PD-1$^+$CD3$^+$CD4$^+$, %	0.598	0.005*
CD3$^+$CD8$^+$, %	0.647	0.002*
Annexin-V$^+$CD3$^+$CD8$^+$, %	0.712	<0.001**
PDL-1$^+$CD3$^+$CD8$^+$, %	0.514	0.020*
PD-1$^+$CD3$^+$CD8$^+$, %	0.267	0.254
CD20$^+$, %	-0.338	0.145
Annexin-V$^+$CD20$^+$, %	-0.249	0.29
PDL-1$^+$CD20$^+$, %	-0.311	0.182
PD-1$^+$CD20$^+$, %	-0.103	0.667

Table S2. Associations between percentages of annexin-V$^+$PD-L1$^+$ and annexin-V$^+$PD-1$^+$ cells and the significant variables in NHL patients. *$p < 0.05$; **$p < 0.001$.

	**Annexin-V$^+$PDL-1$^+$, %**		**Annexin V$^+$PD-1$^+$, %**	
	**r**	***p***	**r**	***p***
Annexin-V$^+$PDL-1$^+$, %	1		0.593	0.006
Annexin-V$^+$PD-1$^+$, %	0.593	0.006*	1	
Annexin-V$^+$, %	0.791	<0.001**	*	0
CD3$^+$, %	0.606	0.005*	0.66	0.002
Annexin-V$^+$CD3$^+$, %	0.678	0.001**	0.707	0
PDL-1$^+$CD3$^+$, %	0.525	0.018*	0.633	0.003
PD-1$^+$CD3$^+$, %	0.715	<0.001**	0.75	0
CD3$^+$CD4$^+$, %	0.609	0.004*	0.515	0.02
Annexin-V$^+$CD3$^+$CD4$^+$, %	0.313	0.179	0.367	0.112
PDL-1$^+$CD3$^+$CD4$^+$, %	-0.014	0.952	-0.301	0.198
PD-1$^+$CD3$^+$CD4$^+$, %	0.673	0.001*	0.283	0.227
CD3$^+$CD8$^+$, %	0.629	0.003*	0.486	0.03
Annexin-V$^+$CD3$^+$CD8$^+$, %	0.646	0.002*	0.53	0.016
PDL-1$^+$CD3$^+$CD8$^+$, %	0.273	0.244	0.376	0.102
PD-1$^+$CD3$^+$CD8$^+$, %	0.363	0.116	0.122	0.609
CD20$^+$, %	-0.307	0.188	-0.352	0.128
Annexin-V$^+$CD20$^+$, %	-0.172	0.468	-0.32	0.169
PDL-1$^+$CD20$^+$, %	-0.318	0.172	-0.433	0.056
PD-1$^+$CD20$^+$, %	-0.079	0.742	-0.131	0.582

Table S3. Comparison of different parameters according to the presence or absence of hepatomegaly and splenomegaly among NHL patients. NHL: Non-Hodgkin lymphoma; N: number.

	**Hepatomegaly**			**Splenomegaly**		
	**Positive (N=7)**	**Negative (N=13)**	***p*-value**	**Positive (N=9)**	**Negative (N=11)**	***p*-value**
PDL-1$^+$CD3$^+$, %	53 (47.6-65)	40.1 (32.7-57.4)	0.383	47.6 (32.7-53)	55 (36-60.4)	0.196
PDL-1$^+$ CD3$^+$CD4$^+$, %	23 (17-25)	19 (16-21)	0.121	21 (20-23)	19 (15-22)	0.093
PDL-1$^+$CD3$^+$CD8$^+$, %	8 (6-11)	8.1 (6-11.4)	0.606	8 (6-9)	8.1 (6-14)	0.402
PDL-1$^+$CD20$^+$, %	17 (14-24)	16 (14-17)	0.127	17 (16-23)	16 (13-17)	0.115
PD-1$^+$ CD3$^+$, %	9 (8-12)	10 (8-11)	0.936	9 (8-10)	10 (8-13)	0.154
PD-1$^+$ CD3$^+$CD4$^+$, %	7 (5-8)	7 (6-7)	0.871	6 (5-7)	7 (6-8)	0.274
PD-1$^+$CD3$^+$CD8$^+$, %	5 (4-6)	5 (4-6)	0.87	5 (4-6)	5 (4-6)	0.844
PD-1$^+$CD20$^+$, %	8 (7-10)	8 (7-9)	0.840	9 (8-9)	8 (7-9)	0.462
Annexin-V$^+$PDL-1$^+$, %	40 (39-52)	40 (39-43)	0.84	40 (39-40)	41 (39-57)	0.215
Annexin-V$^+$PD-1$^+$, %	9 (9-12)	10 (9-11)	0.935	9 (9-9)	11 (9-12)	0.078
Annexin-V$^+$CD3$^+$, %	37 (25-49)	32 (26-42)	0.691	32 (24-37)	40 (32-50)	0.101
Annexin-V$^+$CD3$^+$CD4$^+$, %	33 (21-49)	26 (24-32)	0.218	27 (23-33)	28 (24-45)	0.97
Annexin-V$^+$CD3$^+$CD8$^+$, %	10 (9-13)	11 (9-12)	0.904	10 (9-12)	12 (9-13)	0.218
Annexin-V$^+$CD20$^+$, %	21 (16-24)	17 (16-20)	0.227	21 (17-22)	16 (16-20)	0.246
CD3$^+$, %	60.9 (40-67.6)	55 (44-68.8)	0.782	55 (38-60.9)	65.8 (52-71.2)	0.102
CD3$^+$CD4$^+$, %	36 (30-44)	41 (34-47.5)	0.321	36 (33-44)	41 (32-52)	0.323
CD3$^+$CD8$^+$, %	16 (14-20)	18 (14-22)	0.873	16 (14-20)	20 (14-23)	0.358
CD20$^+$, %	25 (18-27)	21 (19-24)	0.265	25 (22-26)	20 (18-24)	0.194

Table S4. Associations between PD-L1$^+$ cells and the significant variables in NHL patients. *$p < 0.05$.

	**PDL-1$^+$ CD3$^+$, %**	**PDL-1$^+$ CD3$^+$, %**	**PDL-1$^+$ CD4$^+$, %**	**PDL-1$^+$ CD4$^+$, %**	**PDL-1$^+$ CD8$^+$, %**	**PDL-1$^+$ CD8$^+$, %**	**PDL-1$^+$ CD20$^+$, %**	**PDL-1$^+$ CD20$^+$, %**
	**r**	***p***	**r**	***p***	**r**	***p***	**r**	***p***
PDL-1$^+$CD3$^+$CD4$^+$, %	-0.302	0.196						
PDL-1$^+$CD3$^+$CD8$^+$, %	0.21	0.375	-0.436	0.055				
PDL-1$^+$CD20$^+$, %	-0.246	0.296	0.331	0.153	-0.286	0.222		
PD-1$^+$ CD3$^+$, %	0.578	0.008*	-0.007	0.977	0.345	0.136	0.079	-0.401
PD-1$^+$CD3$^+$CD4$^+$, %	0.365	0.113	-0.015	0.95	0.387	0.091	0.826	-0.052
PD-1$^+$CD3$^+$CD8$^+$, %	-0.045	0.851	0.002	0.993	0.012	0.961	0.544	0.144
PD-1$^+$CD20$^+$, %	-0.146	0.54	0.001	0.996	-0.392	0.087	0.373	0.21
CD3$^+$, %	0.472	0.036*	-0.134	0.573	0.596	0.006*	0.236	-0.278
CD3$^+$CD4$^+$, %	0.329	0.157	-0.314	0.178	0.451	0.046*	0.127	-0.353
CD3$^+$CD8$^+$, %	0.261	0.267	-0.338	0.145	0.573	0.008*	0.134	-0.347
CD20$^+$, %	-0.21	0.373	0.273	0.245	-0.324	0.163	0	0.912

Table S5. Associations between PD-1$^+$ cells and the significant variables in NHL patients. *$p < 0.05$.

	**PD-1$^+$CD3$^+$, %**		**PD-1$^+$ CD3$^+$CD4$^+$, %**		**PD-1$^+$ CD3$^+$CD8$^+$, %**		**PD-1$^+$CD20$^+$, %**	
	**r**	***p***	**r**	***p***	**r**	***p***	**r**	***p***
PD-1$^+$ CD3$^+$CD4$^+$, %	0.547	0.013*						
PD-1$^+$ CD3$^+$CD8$^+$, %	0.139	0.559	0.375	0.103				
PD-1$^+$CD20$^+$, %	-0.08	0.738	0.087	0.716	0.625	0.003*		
CD3$^+$, %	0.762	<0.001**	0.58	0.007*	-0.019	0.937	-0.246	0.296
CD3$^+$CD4$^+$, %	0.525	0.018*	0.386	0.093	-0.045	0.849	-0.264	0.261
CD3$^+$CD8$^+$, %	0.454	0.045*	0.513	0.021*	0.424	0.062	-0.045	0.85
CD20$^+$, %	-0.444	0.05*	-0.103	0.665	0.033	0.89	0.084	0.726
